# PATtyFams: Protein Families for the Microbial Genomes in the PATRIC Database

**DOI:** 10.3389/fmicb.2016.00118

**Published:** 2016-02-08

**Authors:** James J. Davis, Svetlana Gerdes, Gary J. Olsen, Robert Olson, Gordon D. Pusch, Maulik Shukla, Veronika Vonstein, Alice R. Wattam, Hyunseung Yoo

**Affiliations:** ^1^Computation Institute, University of ChicagoChicago, IL, USA; ^2^Computing, Environment and Life Sciences, Argonne National LaboratoryArgonne IL, USA; ^3^Fellowship for Interpretation of GenomesBurr Ridge, IL, USA; ^4^Department of Microbiology and Carl R. Woese Institute for Genomic Biology, University of Illinois at Urbana–ChampaignUrbana, IL, USA; ^5^Mathematics and Computer Science Division, Argonne National LaboratoryArgonne, IL, USA; ^6^Virginia Bioinformatics Institute, Virginia Tech UniversityBlacksburg, VA, USA

**Keywords:** genome annotation, comparative genomics, metabolic modeling, FIGfams, RAST

## Abstract

The ability to build accurate protein families is a fundamental operation in bioinformatics that influences comparative analyses, genome annotation, and metabolic modeling. For several years we have been maintaining protein families for all microbial genomes in the PATRIC database (Pathosystems Resource Integration Center, patricbrc.org) in order to drive many of the comparative analysis tools that are available through the PATRIC website. However, due to the burgeoning number of genomes, traditional approaches for generating protein families are becoming prohibitive. In this report, we describe a new approach for generating protein families, which we call PATtyFams. This method uses the k-mer-based function assignments available through RAST (Rapid Annotation using Subsystem Technology) to rapidly guide family formation, and then differentiates the function-based groups into families using a Markov Cluster algorithm (MCL). This new approach for generating protein families is rapid, scalable and has properties that are consistent with alignment-based methods.

## Introduction

The ability to generate accurate protein families is a fundamental component for many bioinformatic applications. It enables evolutionary and contextual comparisons of homologous proteins within and across genomes (Smith, 1990). For instance, genome annotation tools often use protein family data to aid in the propagation of annotations to new genomes (Meyer et al., 2009; Haft et al., 2013; Tatusova et al., 2013a). In metabolic modeling, protein families are often used to help fill gaps in draft models (Henry et al., 2010; Benedict et al., 2014; Seaver et al., 2014). On the PATRIC website, data from protein families are used to drive a variety of comparative analysis tools including the compare regions viewer where users can compare the genomic context of genes, and the heat map display where users can view protein family membership across any set of organisms in the database (Wattam et al., 2014a).

Maintaining up-to-date protein family data for sequenced genomes is challenging because the number of genomes is growing rapidly and traditional methods of family generation are computationally intensive. The most commonly used methods for protein family generation start by using alignment-based tools such as BLAST (Camacho et al., 2009) with a similarity-based threshold in order to determine family membership (Enright et al., 2002; Li et al., 2003; Penel et al., 2009; Punta et al., 2011; Haft et al., 2013; Mi et al., 2013; Galperin et al., 2014). In most cases, rather than doing *ab initio* all vs. all comparisons, sets of representative alignments are maintained for each family and new sequences are added to these representative sets. When a new sequence differs from the preexisting set, it nucleates a new family (e.g., Hobohm et al., 1992; Eddy, 2009). Since aligning sequences can be slow, more recent tools have shifted to using k-mer-based strategies for computing similarity in order to reduce the cost of comparing many sequences (in this case k-mers are short amino acid sequences) (Li and Godzik, 2006; Edgar, 2010; Mahmood et al., 2012; Hauser et al., 2013). Other approaches have reduced computation time by building families for close relatives first, and then subsequently merging the families of more distantly related phylogenetic groups (Halachev et al., 2011).

For several years PATRIC has been providing protein family data that are based on FIGfams (Meyer et al., 2009). FIGfams are protein families that are built from the manually curated annotation data in the SEED database (Overbeek et al., 2005). When an annotator identifies the function of a protein in the literature, they attach it to the protein sequence in the SEED. When possible, collections of related functions called subsystems are built to aid in projecting functions to new genomes. Each function in a subsystem is then used to nucleate a FIGfam. When a new protein matches a representative set of proteins from a given FIGfam, it is considered to be a family member. Providing protein families based on FIGfams is advantageous because they are projections of manual annotations and they can be computed quickly for any set of genomes by k-mer projection (Edwards et al., 2012; Overbeek et al., 2014; Brettin et al., 2015); however, the FIGfam collection only grows as quickly as new annotations are incorporated into subsystems. For the PATRIC project, we wanted protein families that reflect our manual genome annotation efforts, but can also cover all of the proteins in the database.

In this report, we describe a rapid and scalable method for protein family generation that we have designed for the comparative analysis tools on the PATRIC website. Similar to FIGfams, the PATtyFams are based on the standard RAST annotation vocabulary, which is used by the automated metabolic modeling applications in PATRIC, ModelSEED (Henry et al., 2010), and KBase (kbase.us). We describe the PATtyFam algorithm and compare PATtyFams to other protein family generation algorithms.

## Materials and methods

### The algorithm for generating PATtyFams

**Figure 1:**
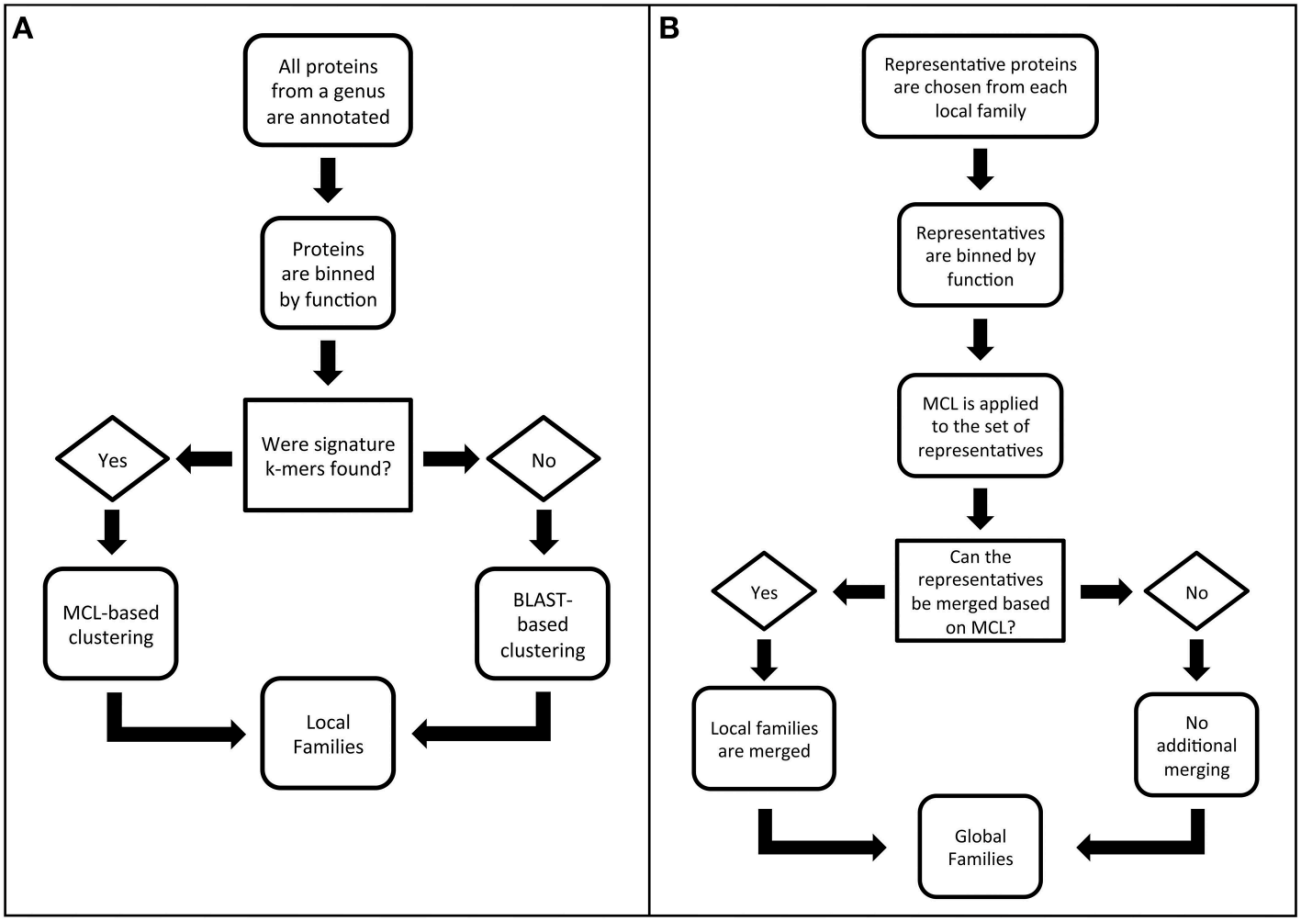
**Flow diagrams outlining the PATtyFam generation procedure for (A) local families and (B) global families**.

The algorithm for generating PATtyFams has three parts. The first part is the computation of local protein families for each genus in PATRIC. The second part is the merger of protein families across genera in order to provide global families. We separated the generation of local and global families because the local families are valuable for many analyses, such as pangenome studies (Tettelin et al., 2005). They also provide a more highly resolved view of protein family membership, which is often lost at greater phylogenetic distances when orthologs and paralogs become difficult to distinguish. The third part is the projection of global family membership to genera with very few sequenced genomes. This third step is also used to compute family membership for new genomes, without having to recompute the entire PATtyFam collection. A flow diagram describing the local and global family generation is shown in Figure 1, and each step is described in detail below.

*Step 1. Local protein families for each genus in PATRIC are computed*. The first part of the PATtyFam computation is the generation of local protein families as described in the steps below.

*Step 1.1. Genomes are binned by genus, pooling identical proteins to make a nonredundant protein database*. The first part of the PATtyFam computation starts by generating genus-level protein families. All of the genomes in PATRIC are binned by genus using the NCBI taxonomy database (Sayers et al., 2009; Benson et al., 2013). Genera with fewer than four genomes typically do not contain enough proteins for clustering and are excluded from the genus-level family generation.

Some of the genera in PATRIC contain thousands of strains, and a large number of the proteins among these strains are identical. To avoid the redundancy of comparing these identical proteins, one representative of each unique protein sequence is kept for the subsequent family computation, and the remaining proteins are reinserted into the family containing the representative copy at the end. This is achieved rapidly through MD5 hashing of the amino acid sequence (Rivest, 1992). Performing this simple step for the genus *Brucella*—currently 475 genomes—results in a 20-fold reduction in the number of protein sequences for comparison.

*Step 1.2. Functional roles are assigned using signature k-mers for the proteins within each genus*. For many years we have been manually annotating genomes for the SEED project and RAST (Overbeek et al., 2005; Aziz et al., 2008). Recently, we have been focusing our manual annotation efforts on a set of 983 representative bacterial and archaeal genomes that we call the CoreSEED in order provide annotation consistency and accuracy spanning a broad diversity of organisms. The second step in PATtyFam generation therefore utilizes a k-mer-based projection of function from the proteins in the CoreSEED, as is also done by the RAST tool kit (Brettin et al., 2015).

In order to rapidly project protein functions from the CoreSEED, we have been using collections of “signature” k-mers. In this case, “signature” k-mers are computed by first finding all of the 8-mer amino acid sequences in each protein. Then the set is reduced to only those k-mers that occur in ≥80% of proteins that have identical functions. We call this set the “signature” k-mers because they are signatures of a particular function. To assign a function to a protein, its signature k-mers are found and its function is based on the SEED function that has the largest number of signature k-mers in common. The use of signature k-mers differs from other k-mer-based clustering methods because in this case, repetitive elements such as conserved motifs and domains typically do not generate signature k-mers since the k-mers characterizing these domains are found in proteins with different functions. This is advantageous for family generation because these repetitive regions are unlikely to contribute to our measure of similarity (described in Step 1.3).

The “classic” version of RAST uses projections that are based on signature k-mers that are generated from the FIGfam collection (Overbeek et al., 2014). In this case, we chose the strictly annotation-based signature k-mer collection from the CoreSEED in an attempt to reduce the influence of the FIGfam collection on the generation of the PATtyFam collection. Unlike the FIGfams, which are typically updated by adding new proteins to previously computed families, the PATtyFam generation always starts from the functions of the CoreSEED proteins. This is important because it helps to prevent errors that result from the accidental merger of unrelated families, which sometimes occurs during FIGfam generation because of chimeric proteins (such as fusions and mobile elements).

After annotating all of the proteins in each genus, we pass a list containing the signature k-mers that were found and the list of proteins containing each signature k-mer to Step 1.3.

*Step 1.3. A pairwise similarity matrix is computed for proteins with the same function*. A pairwise similarity matrix is computed for each set of proteins with the same function within each genus. Our measure of similarity is defined as the number of signature k-mers that are held in common between the pair of proteins divided by the total length of both proteins. This distance measure resembles common distance measuring techniques such as the Sørensen–Dice index, which have been used by other k-mer comparison algorithms (Dice, 1945; Sørensen, 1948; Mahmood et al., 2012). However, we use total protein length in the denominator because the density of signature k-mers can vary within each protein. Note that we do not cross compare proteins with different functions. This helps to keep the number of total comparisons tractable, but also represents a tradeoff in our ability to gather potentially misannotated proteins into the correct family.

*Step 1.4. Markov clustering is performed on each similarity matrix*. Since homologs and paralogs often occur in a set of proteins with the same annotation, it is necessary to attempt to differentiate the members of the set. We do this by using a Markov Cluster (MCL) algorithm, which is a robust clustering method that has been used successfully by previous studies for generating protein families (van Dongen, 2001; Enright et al., 2002; Li et al., 2003). The MCL algorithm has an inflation value parameter that controls the tightness of the clusters. At this step we use an inflation value of 3, which was chosen empirically by manually building and assessing alignments and trees for protein families that were built using different inflation values (data not shown).

*Step 1.5. “Hypothetical proteins” lacking signature k-mers are clustered using BLAST*. All of the proteins that have fewer than 5 signature k-mers (the default RAST cutoff) are annotated as hypothetical proteins. This set is clustered using BLASTP (Camacho et al., 2009) and a similarity-based clustering algorithm that resembles (Hobohm et al., 1992). The clustering works by making the first sequence in the set a representative. Then the next sequence joins the family if it has ≥80% protein sequence identity with the previous representative, otherwise it becomes the representative of a new family. This process is repeated until all of the sequences are clustered. Presumably faster clustering could be achieved by implementing k-mer similarity-based comparison methods at this step, but the fraction of total proteins is small enough that the BLAST operation is currently manageable.

The clusters of proteins from Steps 1.4 and 1.5 represent the local genus-level protein families.

*Step 2. Global protein families are generated*. The second part of the PATtyFam computation is the merger of genus-level families into global families. Similar to the genus-level families, we also use the annotations of protein functions to guide the formation of the global families. Each global family is made by first finding all of the genus-level families with the same function as defined by the RAST and CoreSEED annotation vocabulary. Since the number of proteins in each genus-level family varies, we randomly select up to 10 proteins to represent each genus-level family and combine them to form a single set of representatives, in order to prevent cluster formation that is based upon the genus rather than protein similarity. We chose to randomly select proteins to represent each genus-level family in order to rapidly select a manageable number of proteins for pairwise comparisons. Next, a pairwise distance matrix is computed for the representatives of the set (as described above) and this is passed to MCL for clustering. In this step we use a more inclusive inflation value of 1.1, which was also chosen empirically by comparing alignments and trees built for families using different inflation values (data not shown). Because of the random selection of representatives, we occasionally observe instances where the representatives of a single genus-level family are split into different clusters because the family members that linked them were not chosen as representatives. When this happens, we merge the incorrectly split clusters into a single cluster. Finally, after the clusters are formed for the set of representatives, the remaining members of the genus-level family are added to the appropriate cluster containing their representatives. These fully populated clusters represent the final set of global families.

During the global family generation, the inflation value for MCL must be set lower in order to allow the bridging of families across larger phylogenetic distances. This has a tendency to result in the re-merger of the highly resolved genus-level families that had been computed in Step 1.4. In other words, within-genus paralogs often get merged back into the same global family because they are more similar than orthologs from other genomes (Remm et al., 2001). To avoid complications from this, we retain both the genus-level and global families.

*Step 3. Proteins from underrepresented genera are added to global families*. Since the genera with less than four genomes lack adequate numbers of proteins for *de novo* genus-level family generation, they have been excluded to this point. To determine global family membership for each of their proteins, we first annotate the genome (as in Step 1.2) and then find the corresponding global families with the same function. We then count the number of signature k-mers that are held in common between the protein and the set of representative proteins that were used to build that global family (from Step 2). The protein is then placed into the global family with the largest number of shared signature k-mers. This procedure is also used for determining global protein family membership for new sequences being annotated by RAST and at PATRIC.

### Selection of genomes for analysis

Forty-three representative *Brucella* genomes from Wattam et al. (2014b) were downloaded from PATRIC and used to represent protein family generation for the genus (Table S1). *Escherichia* genomes were selected by first downloading all of the *Escherichia* genomes in PATRIC—2299 at the time. A concatenated alignment of the DNA sequences corresponding to universal genes from Ciccarelli et al. (2006) was generated and a tree was rendered using FastTree with default nucleotide settings (Price et al., 2010). In order to obtain a set of genomes that was similar in size to the *Brucella* set, we selected 38 representative *Escherichia* genomes with the longest branches from the tree (Table S2). We also selected a set of 80 diverse genomes from the NCBI reference genome collection (Tatusova et al., 2013b) (Table S3).

### Comparison of PATtyFams to other family generation methods

Data for the local PATtyFam computations for *Brucella* and *Escherichia* were extracted from local family runs performed on the entire set of *Brucella* and *Escherichia* genomes in the PATRIC database. These local families were compared with families produced by other methods of family generation that were performed directly on the 43 *Brucella* and 38 *Escherichia* genomes. This was done because performing BLAST-based family generation on all *Brucella* and *Escherichia* is computationally intensive. FIGfams (release 60) (Meyer et al., 2009) were generated from the RAST website (Overbeek et al., 2014). Families based on raw k-mer similarity were generated using kClust (Hauser et al., 2013) with the default settings, which cluster proteins to 30% identity. No iterative clustering was performed. BLAST-based families were generated using BLASTP (Camacho et al., 2009) and OrthoMCL (Li et al., 2003), using the FastOrtho package (http://enews.patricbrc.org/fastortho/). We used an inflation value of 1.5 and a BLASTP *e*-value cutoff of 1e-5. The 80 diverse genomes were used as a proxy for global family generation. In all cases the protein families were computed directly on the set of 80 genomes, as described above. For PATtyFams, global families were approximated using the local family algorithm with the global family inflation value of 1.1 for MCL. This allowed us to compare *de novo* runs for each method.

### Estimating runtimes

The *de novo* genus-level family runtimes reported in Table 1 were computed for PATtyFams and OrthoMCL using the FastOrtho package as described above by running both methods sequentially on a machine with an Intel Xeon 2.2 GHz processor and 529GB of memory. For runtime estimations of PATtyFam assignment displayed in Table 2, proteins were drawn randomly from all genomes in PATRIC. For local family estimation, proteins were drawn randomly from all *Escherichia* genomes in PATRIC. Reported runtimes are estimates that are intended to mirror the performance of RAST. Network speed and caching can influence the overall run times.

**Table 1:** **A comparison of runtimes for *de novo* genus-level family generation using OrthoMCL and PATtyFams**.

Genome sets^*^	**Total computation time (seconds)**	
	**OrthoMCL (FastOrtho package)**	**PATtyFams**
43 representative *Brucella* genomes	37,039	932
38 representative *E. coli* genomes	193,916	2473
80 diverse genomes	91,975	9901
All *Brucella* (466 genomes)	Not computed	1764
All *Escherichia* (2707 genomes)	Not computed	46,207

* *Representative genomes used in this study are listed in Tables S1–S3*.

**Table 2:** **Approximate run times for assigning proteins to the current set of PATtyFams**.

**Number of proteins**	**Approximate run time (seconds)**	
	**Local families**	**Global families**
100	0.9	0.8
1000	2.5	2.0
10,000	19.6	13.2
100,000	97.4	42.4
1,000,000	2011.0	1212.0

### Protein comparisons

Protein family content between core protein families (those with proteins from ≥90% of the genomes) was compared using Venny 2.0.2 (Oliveros, 2007). For protein families with more than one member, proteins were compared using BLASTP (Camacho et al., 2009). All pairwise comparisons between family members were computed and the median percent identity was reported for each family. Protein domains were generated by comparison to the NCBI Conserved Domain Database (CDD) (Marchler-Bauer et al., 2014). Unless otherwise stated, domains are reported as matches to “specific” hits. Chromosomal context was computed for each member of a protein family by finding all functions 5 kbp upstream and downstream of the protein encoding gene for each family member and comparing the corresponding sets of functions for each protein in a given family.

## Results

### PATtyfam characteristics

Supporting accurate protein families for all microbial genomes is critical for maintaining a robust comparative analysis infrastructure at PATRIC. In the past we have used BLAST-based methods such as OrthoMCL to generate families, but the computational overhead of all-vs.-all BLAST comparisons has made this infeasible (Li et al., 2003). We have been maintaining FIGfam assignments for all genomes, but these annotation-based families were not designed to cover all proteins (Meyer et al., 2009). In building the PATtyFams, we sought to create a method that captures the annotation consistency of RAST, while incorporating the efficiency of non-alignment-based clustering methods using k-mers (Edgar, 2010; Hauser et al., 2013).

We started by computing local families for all of the bacterial and archaeal genera in PATRIC for which we have a sufficient number of genomes—currently 409 genera. Many factors influence the number of protein families that are formed for each genus, including the number of genomes, evolutionary divergence of strains, genome sizes, horizontal gene transfer events, and nomenclatural boundaries. We observe a large range in the number of families formed per genus with the smallest being 305 local families in *Candidatus Portiera*, bacterial endosymbionts of whiteflies (Jiang et al., 2012), to 247,449 families in *Streptomyces*, soil bacteria that are well known for having very large genomes and diverse secondary metabolic abilities (Bentley et al., 2002). An average of 70% of the local families in each genus are generated by signature k-mers rather than by BLAST comparison, with the lowest coverage by signature k-mers occurring in *Entomoplasma* (with 40% of the families being generated by signature k-mers) and the highest coverage by signature k-mers occurring in *Candidatus Portiera* (with 97% of the families being generated by signature k-mers) (Table S4). After the local families were formed, we merged them across genera in order to generate the set of global families. Overall, 3,935,759 global families were generated for the entire PATRIC database.

The amount of time required to generate PATtyFams for each genus varies, but is much faster than OrthoMCL, which requires an all-vs.-all BLAST comparison. For instance, a *de novo* generation of PATtyFams for 43 representative *Brucella* genomes is ~40 times faster than OrthoMCL, and a *de novo* generation of PATtyFams for 38 representative *Escherichia* genomes is ~80 times faster than OrthoMCL (Table 1). The most time-intensive steps in the PATtyFam algorithm are the k-mer distance computation and the MCL-based family formation, so as the number of new families begins to plateau with the addition of new genomes, the total time required to process each genome decreases. For instance, family generation for the 38 representative *Escherichia* genomes takes ~65 s per genome and family generation for the entire genus (2707 genomes) takes ~17 s per genome (Table 1).

The *de novo* generation of global protein families for the entire PATRIC database currently takes ~2–3 days. Once the entire set is built, the assignment of local and global family membership to the proteins from a new genome is rapid. For a typical genome encoding 5000 proteins, assigning local and global family membership takes ~10 s (Table 2).

### Protein family size and content

We wanted to compare the characteristics of the PATtyFams with other methods of protein family generation, because unlike other methods, PATtyFams use signature k-mers for calling functions, and generating clusters. To do this, we compared them with FIGfams as an example of an annotation-based method, OrthoMCL as an example of a BLAST-based method, and kClust as an example of a k-mer similarity-based method. Although the overarching design goals of each method differ (e.g., building isofunctional homologs, vs. building sets of orthologs, vs. building similarity based clusters), these methods provide a useful benchmark for understanding the characteristics of the PATtyFams. We want to gain an understanding of their inclusivity by comparing the sizes of the families that are made. More specifically, we want to determine if the PATtyFams generate a common set of core families with the other methods.

For local family generation, we examined genomes from *Brucella* and *Escherichia*. We chose *Brucella* because we have extensively annotated these genomes in the past (Wattam et al., 2014b; Faria et al., 2015) and they are a good example of a genus where the sequenced members are closely related. We chose the 43 genomes from Wattam et al. (2014b) to represent the genus (Table S1). We also examined 38 diverse *Escherichia* genomes because they are well studied and are known for having a large amount of horizontally transferred DNA that impacts their phenotypes (Perna et al., 2001) (Table S2). As a proxy for global family generation, we chose to build families for 80 diverse reference genomes taken from the NCBI reference genome collection (Tatusova et al., 2013b) (Table S3).

**Figure 2:**
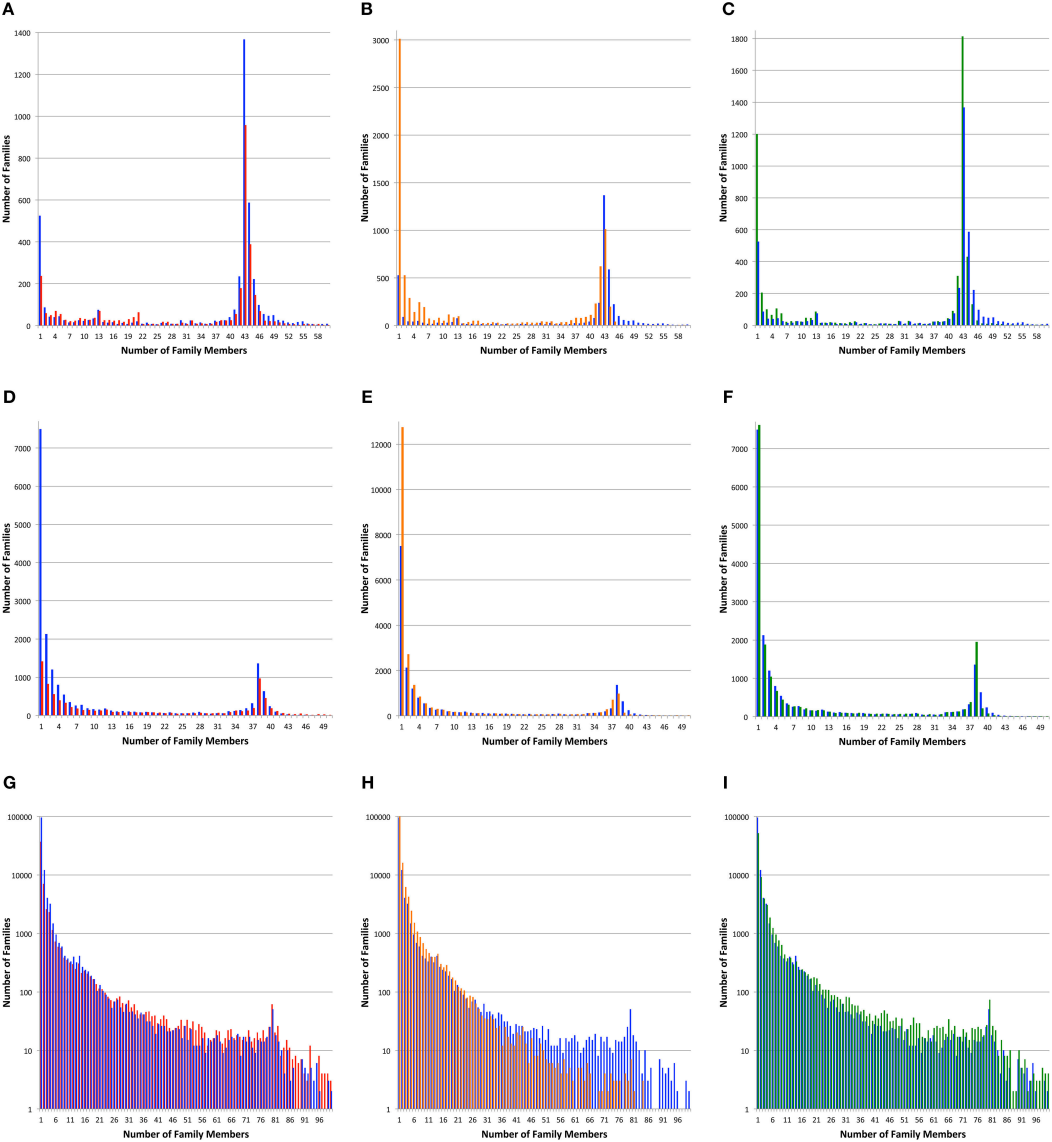
**Protein family sizes**. Histograms depict the number of protein families vs. the number of family members. Protein families were generated with PATtyFams (blue bars), FIGfams (red bars), kClust (orange bars), and OrthoMCL (green bars). The first row **(A–C)** shows families generated for the 43 *Brucella* genomes. The second row **(D–F)** shows families generated for the 38 *Escherichia* genomes. The third row shows families generated for the 80 diverse genomes. Note that the scale of the Y-axis changes and is shown in log-scale for **(G–I)**.

For all three genome sets, we observe that kClust generates the largest number of protein families and that FIGfam assignment generates the fewest (noting that FIGfams were not designed to cover all proteins) (Table 3). For local family generation, OrthoMCL and PATtyFams generate 5340 and 4266 families respectively for *Brucella* and 17,940 and 18,432 families respectively for *Escherichia* (Figure 2). In the case of global family generation, PATtyFams generate more families than OrthoMCL (123,263 vs. 79,013). In all three cases, kClust generates more singleton families than the other methods. For local family generation, PATtyFams generate fewer singleton families than OrthoMCL, but for global family generation PATtyFams generate more singletons than OrthoMCL. For all three genome sets, OrthoMCL generates the largest number of families for which the number of proteins is equal to the number of genomes in the set; however, when you compare the number of families that are generated by each method for which the number of proteins is greater than or equal to the number of genomes in each set, FIGfams and PATtyFams generate more families than OrthoMCL (1956 and 2612 vs. 1516) for *Brucella*, and PATtyFams generate more families than OrthoMCL for *Escherichia* (2477 vs. 2424). This is likely due to OrthoMCL attempting to differentiate paralogs. Although the methods differ greatly in the number of small families that are generated, they yield similar numbers of families that are greater than or equal to the number of genomes in each set (Table 3). These results indicate that PATtyFams are yielding clusters that are comparable in size with other family generation methods, even though they use signature k-mers, rather than alignments or similarity-based k-mers, to create clusters, and they limit the comparison space to proteins with the same functions rather than doing all-vs.-all comparisons.

**Table 3:** **A comparison of PATtyFams to FIGfams, kClust, and OrthoMCL**.

	**FIGfams**	**kClust**	**OrthoMCL**	**PATtyFams**
**LOCAL FAMILIES FOR** ***Brucella*** **(43 GENOMES)**				
Total Families	3407	8182	5340	4266
Families with >43 members	1000	372	704	1246
Families with 43 members	956	1010	1812	1366
Families with < 43 members	1451	6800	2824	1654
Families with one member	236	3009	1200	524
**LOCAL FAMILIES FOR** ***Escherichia*** **(38 GENOMES)**				
Total Families	8681	24,046	17,940	18,432
Families with >38 members	1297	638	477	1124
Families with 38 members	961	970	1947	1353
Families with < 38 members	6423	22,438	15,516	15,955
Families with one member	1411	12,744	7611	7487
**GLOBAL FAMILIES (80 DIVERSE GENOMES)**				
Total Families	57,147	137,785	79,013	123,263
Families with >80 members	329	24	219	158
Families with 80 members	61	7	73	50
Families with < 80 members	56,757	137,754	78,721	123,055
Families with one member	36,700	99,876	51,582	94,844

**Figure 3:**
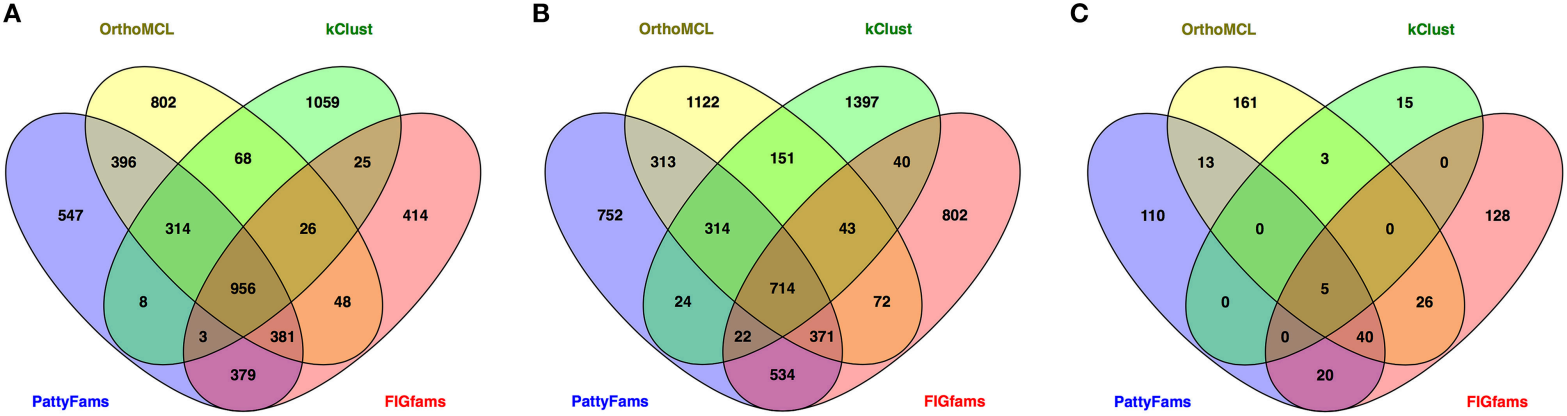
**Venn diagrams showing the number of identical protein families held in common between PATtyFams (blue), OrthoMCL (yellow), kClust (green), and FIGfams (red) for (A) the 43 *Brucella* genomes, (B) the 38 *Escherichia* genomes, and (C) the 80 diverse genomes**. Data are shown for core protein families, defined as those families that have proteins from ≥90% of genomes in each set.

We also wanted to compare the content of the protein families generated by each method. Since the number of small families varies considerably, we chose to focus on the core set of protein families—those that contain a protein from =90% of the genomes in each set. We then searched for families that were identical between each method (Figure 3). Overall for the local families, PATtyFams have the most proteins families in common with other methods: 2437 vs. 2189, 1400, and 1818 for *Brucella;* and 2292 vs. 1978, 1308, and 1796 for *Escherichia* for OrthoMCL, kClust, and FIGfams respectively. For the global families, PATtyFams share fewer protein families (78) than OrthoMCL (87) or FIGfams (91), but more than kClust (8). PATtyFams also tend to have a smaller number of idiosyncratic families that are not identical with those generated by the other methods. For the local families, PATtyFams have the most families in common with OrthoMCL; for the global families, PATtyFams have two more families in common with FIGfams (60) than OrthoMCL (58). Overall, the core protein content of the PATtyFams is consistent with other methods, and more closely resembles OrthoMCL and FIGfams than kClust. PATtyFams also appear to be advantageous because they find the set of shared core families but generate few idiosyncratic core families.

### Protein similarity within families

**Figure 4:**
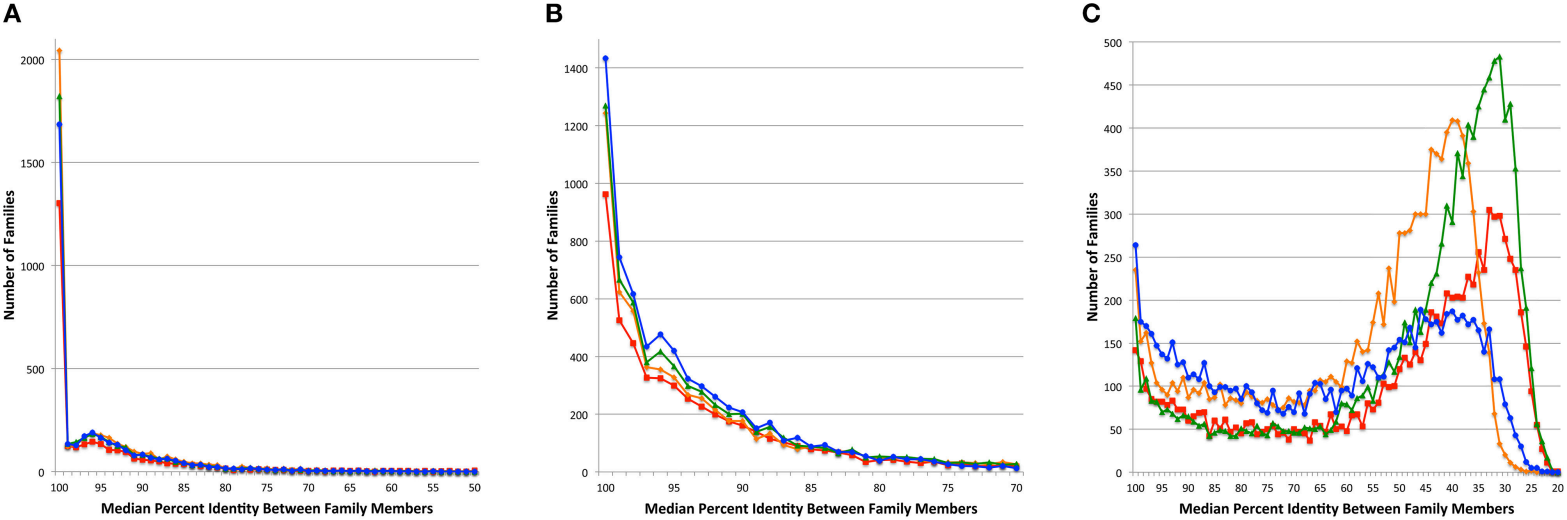
**Median percent identity among family members**. Histograms depict the number of protein families vs. the median percent identity for all pairwise BLAST comparisons between family members for **(A)** the 43 *Brucella* genomes, **(B)** the 38 *Escherichia* genomes, and **(C)** the 80 diverse genomes. Families generated by FIGfams are depicted as red lines with square plot points, kClust are orange lines with diamond plot points, OrthoMCL are green lines with triangle plot points, and PATtyFams are blue lines with circle plot points.

Since the PATtyFams are based upon signature k-mers rather than protein similarity *per se*, we wanted to perform a BLAST comparison of the protein family members in order to measure the similarity among members of a given protein family. For each protein family, we performed all pairwise BLASTP comparisons measuring the median percent identity for the comparison of all family members for PATtyFams (Camacho et al., 2009). We then compared this to the same analysis for FIGfams, kClust and OrthoMCL (Figure 4). For *Brucella*, the median percent identity for comparisons is nearly the same for all protein family methods. OrthoMCL and kClust appear to have slightly more families with 100% median identity, but this may be due to the larger number of small families made by both methods (Figure 2). For *Escherichia*, PATtyFams have a larger number of families with 100% median identity and slightly more families with median percent identities >90%. In the case of the global families, PATtyFams have slightly more families with >80% identity, and dramatically fewer families with low median percent identities between 50 and 20%. Overall, the signature k-mer based PATtyFams are consistent when the members are compared with BLAST, and they are stricter than other methods at excluding lower similarity proteins during the global merging process.

**Figure 5:**
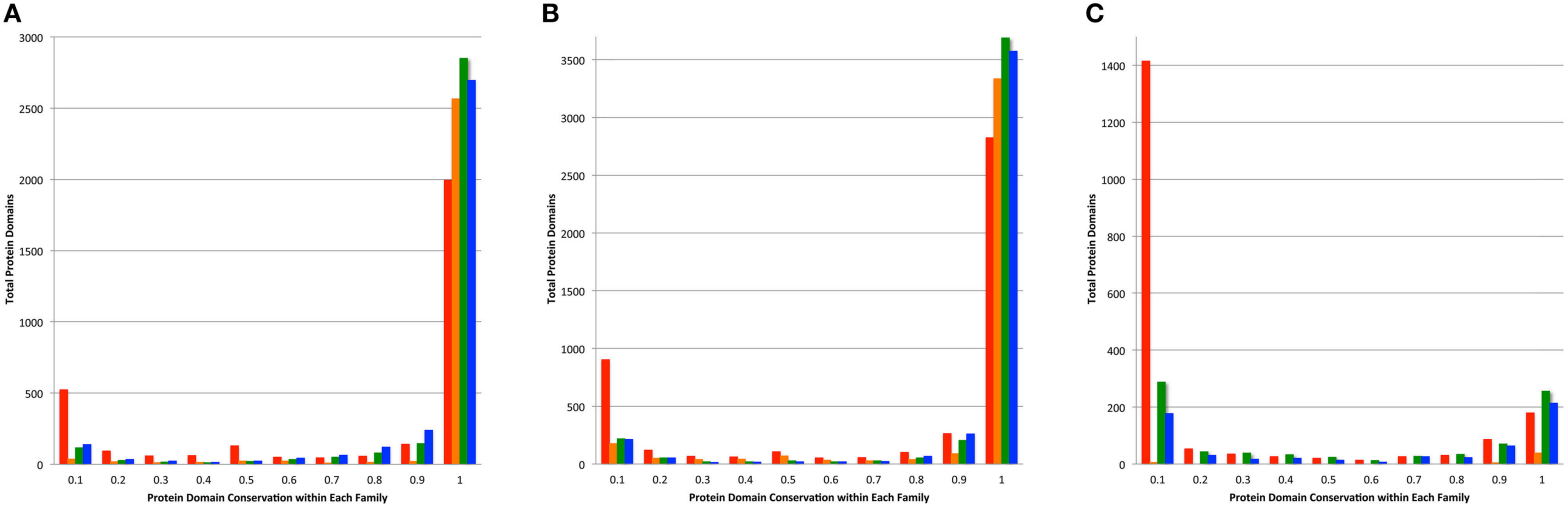
**Conservation of protein domains within family members**. Histograms depict the total number of protein domains vs. their conservation across all members of each family as generated by FIGfams (red), kClust (orange), OrthoMCL (green), and PATtyFams (blue). Data are shown for the subset of families in which ≥ 90% of the genomes are represented for **(A)** the 43 *Brucella* genomes, **(B)** the 38 *Escherichia* genomes, and **(C)** the 80 diverse genomes.

The conservation of protein domains among the members of a protein family can be an indication of consistency of a family because infrequently occurring protein domains can indicate the presence of fragmented proteins and protein fusions. For each protein in a family generated by PATtyFams, OrthoMCL, kClust, and FIGfams, we found the protein domains by comparing each protein to the NCBI CDD (Marchler-Bauer et al., 2014). In order to avoid small fragmented clusters influencing the analysis, we examined protein domain conservation in the set of protein families that are represented by at least 90% of the genomes in each set (Table S5). PATtyFams have slightly fewer families than OrthoMCL in the set of protein families that have 100% conservation among all members (Figure 5). The number of families with domain conservation < 100% is nearly identical between PATtyFams and Ortho MCL, except in the 80 diverse genomes set, where the PATtyFams have fewer families in the bin with =10% conservation of a protein family (Figure 5C). Overall, PATtyFams are most similar to OrthoMCL in protein domain conservation between family members.

### Conservation of chromosomal context among family members

**Figure 6:**
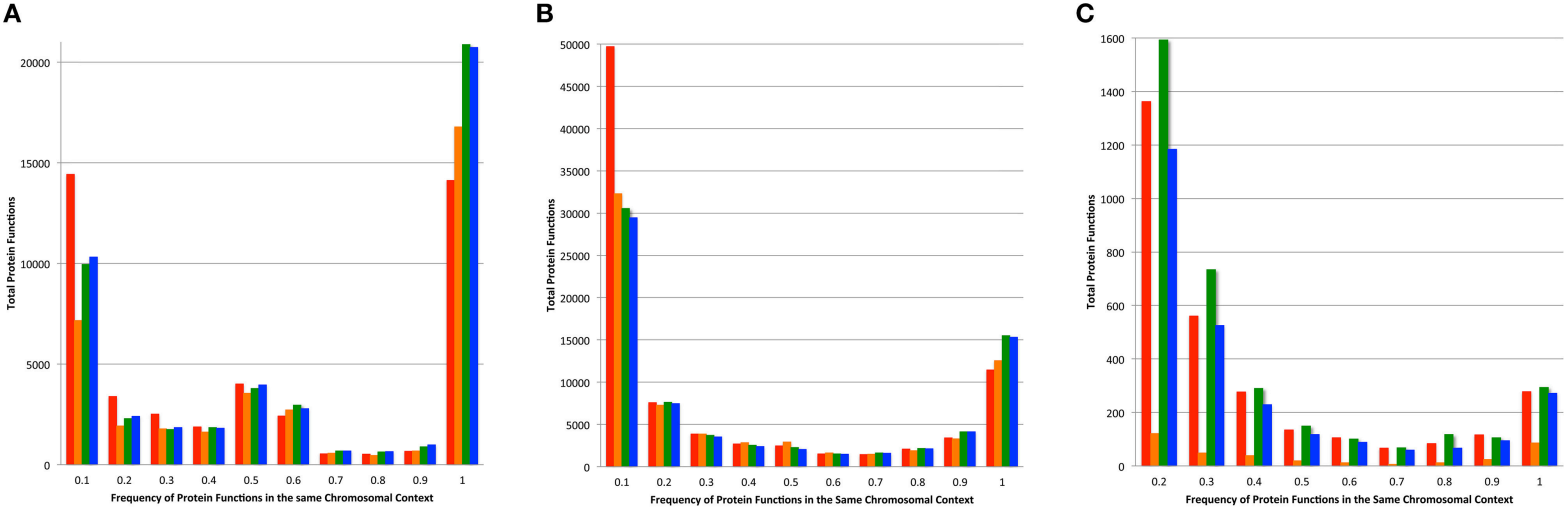
**Chromosomal context conservation within family members**. For the protein-encoding gene of each family member, the functions of its neighboring genes 5 kbp upstream, and downstream were obtained. Histograms depict the total number of functions vs. their conservation among family members. Data for families generated by FIGfams are shown in red, kClust are orange, OrthoMCL are green, and PATtyFams are blue. Data are shown for the subset of protein families in which ≥90% of the genomes are represented for **(A)** the 43 *Brucella* genomes, **(B)** the 38 *Escherichia* genomes, and **(C)** the 80 diverse genomes. Note that the number of proteins in the 0.1 bin is not displayed for the 80 diverse genomes and is 96,117 for FIGfams, 5540 for kClust, 75,070 for OrthoMCL, and 55,525 for PATtyFams.

In most microbial genomes there is strong conservation in the chromosomal context of protein encoding genes across phylogenetic distances. This provides the bedrock for comparative analysis and the projection of protein functions (Overbeek et al., 1999, 2005; Davis et al., 2014). We compared the functions of proteins found 5 kbp upstream and downstream of each protein in a family, and compared this set of nearby functions among family members (Figure 6). As above, we performed this analysis on the set of core proteins to prevent the influence of fragmented families on the analysis. Overall, the PATtyFams track very closely with OrthoMCL, having slightly fewer families with 100% chromosomal conservation in all three cases. In the other bins, PATtyFams track closely with OrthoMCL except for the 80 diverse genomes, where the PATtyFams generate dramatically fewer proteins families with aberrant chromosomal contexts (Figure 6C). This may indicate a better resolution of paralogs, protein fusions or protein fragments, or that the inclusion criterion for global family membership is simply stricter.

### Availability

The current version of PATtyFams is available for browsing on the PATRIC website (www.patricbrc.org) where they can be used to drive the comparative analysis tools. PATtyFams have also been computed for all genomes in PATRIC and can be downloaded with each genome from the FTP site (e.g., ftp://ftp.patricbrc.org/patric2/patric3/genomes/83332.12/83332.12.PATRIC.cds.tab, where 833332.12 is an example genome ID). The RASTtk version of RAST (rast.nmpdr.org) and the annotation service on the PATRIC website can both be used to compute PATtyFam membership for the proteins in a genome. The command line script for generating PATtyFams, *rast-annotate-families-patric*, has also been distributed in the RAST tool kit (https://github.com/TheSEED/RASTtk-Distribution).

## Discussion

We have created an annotation-based method for generating protein families that is scalable and provides rapid protein family assignments locally at the genus level and globally across all genomes. Unlike other methods, PATtyFams are not built from all-vs.-all comparisons, instead utilizing the annotation data from RAST to form the initial clusters and the signature k-mer data associated with each protein to differentiate the clusters. The data presented in this report suggest that this approach is robust and accurate.

In this study, we compared PATtyFams to other commonly used protein family generation methods. It is difficult to objectively assess whether one method of protein family generation is superior to another because each method was designed for a different purpose. Furthermore, depending on the circumstances, one may wish to have very tight protein clusters and another may wish to have clusters that are very inclusive. Nevertheless, we observe that the PATtyFams are consistent with these other methods and tend to have characteristics that are most similar to families generated by OrthoMCL. OrthoMCL creates more bins that are equal to the number of the genomes in the set, while PATtyFams can create bins that are somewhat larger. This is likely due to OrthoMCL attempting to distinguish “recent” paralogs, which PATtyFam algorithm does not do. PATtyFams also share the most identical core local families with other methods, with the largest subset being held in common with OrthoMCL. The median percent identity, conservation of protein domains, and conservation of chromosomal context among family members also most closely resembles OrthoMCL.

The comparison of global families indicates that PATtyFams are more strongly conserved in median percent identity having dramatically fewer proteins with percent identities below 40%. We consider this to be a favorable behavior because families with < 40% identity among members are likely to be inaccurate (Rost, 1999). This is probably the result of the annotation data limiting initial cluster formation. Presumably this behavior could also be achieved by raising the similarity threshold or inflation value for kClust and OrthoMCL, but PATtyFams have this natural behavior in the presence of potentially binnable low similarity sequences. Similar to percent identity, the global families also have fewer core family members with aberrant protein domains and dramatically fewer core family members with aberrant chromosomal contexts. Although this indicates a tighter clustering behavior for global family generation, we consider this to be a favorable behavior as well. We conclude therefore, that PATtyFams method is valuable for binning isofunctional homologs.

This project has enabled us to make improvements in several important comparative analysis tools on the PATRIC website. These include the compare regions tool which allows users to compare the chromosomal context of protein-encoding genes across phylogenetic distances, the protein family sorter which allows users to browse and compare protein family members and to select protein sets for making alignment and trees, and the heat map display of protein family membership which allows users to visually compare genomes and locate horizontally transferred regions. When a user uploads a new genome to the PATRIC annotation service, local and global PATtyFams are automatically computed enabling an integrated contextual view of each genome through the website tools. PATRIC has also recently released a service that enables automated metabolic model reconstruction that is similar to that in the KBase (kbase.us) and ModelSEED (Henry et al., 2010) resources. We anticipate that the ability to build automated metabolic models coupled with the added curation advantage of having comprehensive annotation-based protein families will be beneficial to the modeling community.

## Author contributions

JD, Algorithm design, data analysis, wrote, and prepared manuscript. SG, Data analysis. GO, Algorithm design. RO, Algorithm design; data analysis; manuscript preparation; PATtyFam computation, installation, and distribution; software engineering. GP, Algorithm design. MS, Algorithm design; data analysis; manuscript preparation; PATtyFam computation, installation, and distribution. VV, Data analysis. AW, Data analysis. HY, PATtyFam computation, installation, and distribution.

## Funding

This work was supported by the United States National Institute of Allergy and Infectious Diseases, National Institutes of Health, Department of Health and Human Service [Contract No. HHSN272201400027C]. GO's contributions were supported in part by the NIH [Contract HHSN266200400042C] via a subcontract from the University of Chicago, and by the National Aeronautics and Space Administration through the NASA Astrobiology Institute under Cooperative Agreement No. NNA13AA91A issued through the Science Mission Directorate.

### Conflict of interest statement

The authors declare that the research was conducted in the absence of any commercial or financial relationships that could be construed as a potential conflict of interest.
