# Supplementary material for: PATtyFams: Protein Families for the Microbial Genomes in the PATRIC Database
Source: Front Microbiol. 2016 Feb 8;7:118. doi: 10.3389/fmicb.2016.00118 (PMC4744870; doi:10.3389/fmicb.2016.00118)
Supplement: Supplementary file 1 [file Table1.DOCX]

**Table S1.** The *Brucella* genomes used in this study.

| Genome ID | Genome Name |
| --- | --- |
| 235.15 | Brucella abortus |
| 235.16 | Brucella abortus |
| 235.17 | Brucella abortus |
| 235.18 | Brucella abortus |
| 235.19 | Brucella abortus |
| 235.20 | Brucella abortus |
| 235.21 | Brucella abortus |
| 1104320.3 | Brucella abortus A13334 |
| 262698.4 | Brucella abortus bv. 1 str. 9-941 |
| 430066.4 | Brucella abortus S19 |
| 36855.3 | Brucella canis |
| 36855.4 | Brucella canis |
| 483179.4 | Brucella canis ATCC 23365 |
| 1104321.3 | Brucella canis HSK A52141 |
| 1408887.3 | Brucella canis str. Oliveri |
| 520459.3 | Brucella ceti M644/93/1 |
| 1423891.3 | Brucella ceti TE10759-12 |
| 1407053.3 | Brucella ceti TE28753-12 |
| 546272.3 | Brucella melitensis ATCC 23457 |
| 359391.4 | Brucella melitensis biovar Abortus 2308 |
| 224914.11 | Brucella melitensis bv. 1 str. 16M |
| 941967.3 | Brucella melitensis M28 |
| 703352.4 | Brucella melitensis M5-90 |
| 1029825.4 | Brucella melitensis NI |
| 568815.3 | Brucella microti CCM 4915 |
| 520456.3 | Brucella neotomae 5K33 |
| 444178.3 | Brucella ovis ATCC 25840 |
| 120576.3 | Brucella pinnipedialis |
| 520461.7 | Brucella pinnipedialis B2/94 |
| 520448.3 | Brucella sp. NVSL 07-0026 |
| 29461.1 | Brucella suis |
| 29461.11 | Brucella suis |
| 29461.12 | Brucella suis |
| 29461.9 | Brucella suis |
| 204722.15 | Brucella suis 1330 |
| 204722.5 | Brucella suis 1330 |
| 470137.4 | Brucella suis ATCC 23445 |
| 645170.27 | Brucella suis bv. 2 |
| 645170.28 | Brucella suis bv. 2 |
| 645170.29 | Brucella suis bv. 2 |
| 645170.3 | Brucella suis bv. 2 |
| 645170.31 | Brucella suis bv. 2 |
| 1112912.3 | Brucella suis VBI22 |
