# Supplementary material for: PATtyFams: Protein Families for the Microbial Genomes in the PATRIC Database
Source: Front Microbiol. 2016 Feb 8;7:118. doi: 10.3389/fmicb.2016.00118 (PMC4744870; doi:10.3389/fmicb.2016.00118)
Supplement: Supplementary file 2 [file Table2.DOCX]

**Table S2.** The *Escherichia* genomes used in this study.

| Genome ID | Genome Name |
| --- | --- |
| 502347.3 | Escherichia albertii TW07627 |
| 216592.3 | Escherichia coli 042 |
| 1411693.3 | Escherichia coli 104701 |
| 1444039.3 | Escherichia coli 2-156-04_S1_C1 |
| 1444073.3 | Escherichia coli 2-460-02_S1_C2 |
| 869684.5 | Escherichia coli 5.0959 |
| 1444046.3 | Escherichia coli 5-172-05_S1_C1 |
| 1268996.3 | Escherichia coli 90854101 |
| 1269002.3 | Escherichia coli 90863201 |
| 1333592.3 | Escherichia coli AB43739056-isolate1 |
| 405955.13 | Escherichia coli APEC O1 |
| 550674.3 | Escherichia coli B093 |
| 1328435.3 | Escherichia coli BWH 2401 |
| 1126909.3 | Escherichia coli C586_05 |
| 1331061.3 | Escherichia coli E1777 |
| 656390.3 | Escherichia coli H263 |
| 1281098.3 | Escherichia coli HVH 167 (4-6073565) |
| 1432557.3 | Escherichia coli ISC11 |
| 1169321.3 | Escherichia coli KTE114 |
| 1182732.3 | Escherichia coli KTE159 |
| 1169349.3 | Escherichia coli KTE31 |
| 1169351.3 | Escherichia coli KTE33 |
| 1182659.4 | Escherichia coli KTE52 |
| 749546.3 | Escherichia coli MS 185-1 |
| 749528.3 | Escherichia coli MS 45-1 |
| 749532.3 | Escherichia coli MS 78-1 |
| 1446583.3 | Escherichia coli O145:H25 str. 07-3858 |
| 155864.8 | Escherichia coli O157:H7 EDL933 |
| 1446637.3 | Escherichia coli O157:H7 str. 2011EL-2106 |
| 702432.3 | Escherichia coli O157:H7 str. EC4084 |
| 702433.3 | Escherichia coli O157:H7 str. EC4127 |
| 1446703.3 | Escherichia coli O174:H21 str. 03-3269 |
| 1232150.3 | Escherichia coli O26:H11 str. CFSAN001629 |
| 1116075.3 | Escherichia coli P0302293.4 |
| 511145.12 | Escherichia coli str. K-12 substr. MG1655 |
| 1281275.3 | Escherichia coli UMEA 3899-1 |
| 585054.5 | Escherichia fergusonii ATCC 35469 |
| 1115512.3 | Escherichia hermannii NBRC 105704 |
