# Supplementary material for: PATtyFams: Protein Families for the Microbial Genomes in the PATRIC Database
Source: Front Microbiol. 2016 Feb 8;7:118. doi: 10.3389/fmicb.2016.00118 (PMC4744870; doi:10.3389/fmicb.2016.00118)
Supplement: Supplementary file 3 [file Table3.DOCX]

**Table S3.** The diverse genomes used in this study.

| Genome ID | Genome Name |
| --- | --- |
| 176299.1 | Agrobacterium tumefaciens str. C58 |
| 749927.5 | Amycolatopsis mediterranei U32 |
| 198094.11 | Bacillus anthracis str. Ames |
| 260799.14 | Bacillus anthracis str. Sterne |
| 226900.8 | Bacillus cereus ATCC 14579 |
| 224308.43 | Bacillus subtilis subsp. subtilis str. 168 |
| 281309.8 | Bacillus thuringiensis serovar konkukian str. 97-27 |
| 226186.12 | Bacteroides thetaiotaomicron VPI-5482 |
| 702459.3 | Bifidobacterium bifidum PRL2010 |
| 206672.9 | Bifidobacterium longum NCC2705 |
| 257313.5 | Bordetella pertussis Tohama I |
| 224326.49 | Borrelia burgdorferi B31 |
| 224911.5 | Bradyrhizobium japonicum USDA 110 |
| 107806.1 | Buchnera aphidicola str. APS (Acyrthosiphon pisum) |
| 272560.6 | Burkholderia pseudomallei K96243 |
| 192222.6 | Campylobacter jejuni subsp. jejuni NCTC 11168 |
| 190650.5 | Caulobacter crescentus CB15 |
| 471472.4 | Chlamydia trachomatis 434/Bu |
| 272561.5 | Chlamydia trachomatis D/UW-3/CX |
| 115713.3 | Chlamydophila pneumoniae CWL029 |
| 324602.8 | Chloroflexus aurantiacus J-10-fl |
| 272562.8 | Clostridium acetobutylicum ATCC 824 |
| 441771.6 | Clostridium botulinum A str. Hall |
| 272563.8 | Clostridium difficile 630 |
| 196627.14 | Corynebacterium glutamicum ATCC 13032 |
| 227377.7 | Coxiella burnetii RSA 493 |
| 243230.17 | Deinococcus radiodurans R1 |
| 882.5 | Desulfovibrio vulgaris str. Hildenborough |
| 716541.4 | Enterobacter cloacae subsp. cloacae ATCC 13047 |
| 226185.9 | Enterococcus faecalis V583 |
| 585057.6 | Escherichia coli IAI39 |
| 1133852.3 | Escherichia coli O104:H4 str. 2011C-3493 |
| 386585.9 | Escherichia coli O157:H7 str. Sakai |
| 685038.3 | Escherichia coli O83:H1 str. NRG 857C |
| 511145.12 | Escherichia coli str. K-12 substr. MG1655 |
| 585056.7 | Escherichia coli UMN026 |
| 402612.5 | Flavobacterium psychrophilum JIP02/86 |
| 177416.18 | Francisella tularensis subsp. tularensis SCHU S4 |
| 190304.8 | Fusobacterium nucleatum subsp. nucleatum ATCC 25586 |
| 243231.5 | Geobacter sulfurreducens PCA |
| 71421.8 | Haemophilus influenzae Rd KW20 |
| 85962.8 | Helicobacter pylori 26695 |
| 1125630.4 | Klebsiella pneumoniae subsp. pneumoniae HS11286 |
| 272621.13 | Lactobacillus acidophilus NCFM |
| 220668.9 | Lactobacillus plantarum WCFS1 |
| 362948.14 | Lactobacillus salivarius UCC118 |
| 272623.7 | Lactococcus lactis subsp. lactis Il1403 |
| 189518.3 | Leptospira interrogans serovar Lai str. 56601 |
| 169963.11 | Listeria monocytogenes EGD-e |
| 265311.5 | Mesoplasma florum L1 |
| 233413.5 | Mycobacterium bovis AF2122/97 |
| 272631.5 | Mycobacterium leprae TN |
| 246196.19 | Mycobacterium smegmatis str. MC2 155 |
| 83332.12 | Mycobacterium tuberculosis H37Rv |
| 272632.4 | Mycoplasma mycoides subsp. mycoides SC str. PG1 |
| 272634.6 | Mycoplasma pneumoniae M129 |
| 242231.1 | Neisseria gonorrhoeae FA 1090 |
| 122586.8 | Neisseria meningitidis MC58 |
| 167539.5 | Prochlorococcus marinus subsp. marinus str. CCMP1375 |
| 208964.12 | Pseudomonas aeruginosa PAO1 |
| 272943.9 | Rhodobacter sphaeroides 2.4.1 |
| 243090.15 | Rhodopirellula baltica SH 1 |
| 269796.9 | Rhodospirillum rubrum ATCC 11170 |
| 272947.5 | Rickettsia prowazekii str. Madrid E |
| 99287.12 | Salmonella enterica subsp. enterica serovar Typhimurium str. LT2 |
| 220341.7 | Salmonella enterica subsp. enterica serovar Typhi str. CT18 |
| 211586.12 | Shewanella oneidensis MR-1 |
| 300267.13 | Shigella dysenteriae Sd197 |
| 198214.7 | Shigella flexneri 2a str. 301 |
| 93061.5 | Staphylococcus aureus subsp. aureus NCTC 8325 |
| 171101.6 | Streptococcus pneumoniae R6 |
| 568814.3 | Streptococcus suis BM407 |
| 100226.15 | Streptomyces coelicolor A3(2) |
| 243274.5 | Thermotoga maritima MSB8 |
| 300852.9 | Thermus thermophilus HB8 |
| 243277.26 | Vibrio cholerae O1 biovar El Tor str. N16961 |
| 312309.11 | Vibrio fischeri ES114 |
| 190485.4 | Xanthomonas campestris pv. campestris str. ATCC 33913 |
| 393305.7 | Yersinia enterocolitica subsp. enterocolitica 8081 |
| 214092.21 | Yersinia pestis CO92 |
