# Supplementary material for: PATtyFams: Protein Families for the Microbial Genomes in the PATRIC Database
Source: Front Microbiol. 2016 Feb 8;7:118. doi: 10.3389/fmicb.2016.00118 (PMC4744870; doi:10.3389/fmicb.2016.00118)
Supplement: Supplementary file 4 [file Table4.DOCX]

**Table S4.** Characteristics of local PATtyFams computed for each genus in PATRIC.

| Genus | Genomes | Total PATtyFams | Families of hypothetical proteins (generated by BLAST) |
| --- | --- | --- | --- |
| Acetobacter subgen. Acetobacter | 4 | 5136 | 1209 |
| Acholeplasma | 7 | 5293 | 2190 |
| Achromobacter | 17 | 15653 | 3031 |
| Acidaminococcus | 6 | 3704 | 810 |
| Acidiphilium | 5 | 6201 | 1340 |
| Acidithiobacillus | 10 | 10749 | 3922 |
| Acidovorax | 15 | 18874 | 4472 |
| Acinetobacter | 1211 | 55563 | 20661 |
| Actinoalloteichus | 4 | 6355 | 1524 |
| Actinobacillus | 30 | 5816 | 1005 |
| Actinobaculum | 6 | 4951 | 1724 |
| Actinomadura | 6 | 21586 | 6465 |
| Actinomyces | 48 | 24818 | 8094 |
| Actinoplanes | 9 | 29378 | 9679 |
| Actinopolyspora | 4 | 9892 | 3120 |
| Aerococcus | 4 | 3379 | 377 |
| Aeromonas | 83 | 20065 | 6166 |
| Afipia | 15 | 14118 | 3861 |
| Aggregatibacter | 25 | 4906 | 1071 |
| Agrobacterium | 23 | 25486 | 7441 |
| Alcaligenes | 6 | 6167 | 1199 |
| Alcanivorax | 11 | 11443 | 2969 |
| Algoriphagus | 6 | 11026 | 3649 |
| Alicyclobacillus | 9 | 12868 | 5216 |
| Aliivibrio | 7 | 7155 | 898 |
| Alistipes | 11 | 13854 | 4882 |
| Alteromonas | 20 | 12113 | 4099 |
| Amycolatopsis | 26 | 44741 | 12631 |
| Anabaena | 4 | 12278 | 3534 |
| Anaerococcus | 13 | 7373 | 1697 |
| Anaeromyxobacter | 4 | 7021 | 1329 |
| Anaplasma | 24 | 3983 | 1390 |
| Anoxybacillus | 14 | 7923 | 2556 |
| Aquimarina | 7 | 13696 | 4565 |
| Archaeoglobus | 5 | 5419 | 1951 |
| Arcobacter | 12 | 6790 | 1287 |
| Arenimonas | 7 | 6234 | 1454 |
| Arthrobacter | 43 | 38522 | 13765 |
| Arthrospira | 4 | 6943 | 1185 |
| Asaia | 5 | 6563 | 2458 |
| Asticcacaulis | 7 | 12178 | 3555 |
| Atopobium | 14 | 7304 | 2132 |
| Azospirillum | 8 | 19727 | 5082 |
| Azotobacter | 4 | 5919 | 646 |
| Bacillus | 585 | 186454 | 79937 |
| Bacteriovorax | 5 | 9811 | 4369 |
| Bacteroides | 188 | 70230 | 27165 |
| Bartonella | 75 | 13735 | 7875 |
| Bdellovibrio | 6 | 7649 | 2534 |
| Bibersteinia | 4 | 2477 | 134 |
| Bifidobacterium | 167 | 30321 | 11688 |
| Blattabacterium | 8 | 841 | 113 |
| Blautia | 15 | 22680 | 7298 |
| Bordetella | 139 | 19138 | 4183 |
| Borrelia | 115 | 6893 | 2335 |
| Brachybacterium | 5 | 7653 | 1939 |
| Brachyspira | 13 | 6886 | 1670 |
| Bradyrhizobium | 72 | 74273 | 31759 |
| Branhamella | 12 | 2596 | 405 |
| Brevibacillus | 19 | 23885 | 7505 |
| Brevibacterium | 8 | 10208 | 2842 |
| Brevundimonas | 11 | 11012 | 2718 |
| Brucella | 466 | 6557 | 1677 |
| Buchnera | 18 | 1122 | 291 |
| Burkholderia | 317 | 130028 | 51607 |
| Butyrivibrio | 41 | 32915 | 10526 |
| Caldicellulosiruptor | 10 | 6320 | 908 |
| Calothrix | 4 | 16409 | 5766 |
| Campylobacter | 227 | 22989 | 8471 |
| Candidatus Accumulibacter | 9 | 14148 | 4595 |
| Candidatus Arthromitus | 12 | 1870 | 210 |
| Candidatus Blochmannia | 4 | 859 | 122 |
| Candidatus Carsonella | 6 | 472 | 100 |
| Candidatus Liberibacter | 9 | 2740 | 322 |
| Candidatus Pelagibacter | 13 | 4247 | 1224 |
| Candidatus Phytoplasma | 14 | 2831 | 830 |
| Candidatus Portiera | 4 | 305 | 10 |
| Candidatus Sulcia | 8 | 387 | 33 |
| Capnocytophaga | 27 | 15521 | 4879 |
| Carnobacterium | 17 | 11852 | 3539 |
| Caulobacter | 16 | 15488 | 4145 |
| Cedecea | 5 | 7866 | 1031 |
| Cellulomonas | 11 | 14357 | 3338 |
| Cellulophaga | 10 | 9316 | 1236 |
| Chlamydia | 146 | 8646 | 3094 |
| Chlamydophila | 4 | 1332 | 297 |
| Chlorobium | 6 | 7137 | 2050 |
| Chloroflexus | 5 | 5883 | 1331 |
| Chromobacterium | 7 | 9413 | 1895 |
| Chryseobacterium | 38 | 35192 | 12999 |
| Citrobacter | 32 | 16799 | 4774 |
| Clavibacter | 5 | 5080 | 966 |
| Clostridium | 190 | 128695 | 48565 |
| Cohnella | 4 | 10778 | 4051 |
| Collinsella | 7 | 5767 | 1766 |
| Colwellia | 5 | 10647 | 2798 |
| Comamonas | 24 | 18364 | 5043 |
| Coprobacillus | 5 | 6648 | 1165 |
| Coprococcus | 6 | 9504 | 2271 |
| Corynebacterium | 142 | 46252 | 15616 |
| Coxiella | 13 | 2823 | 505 |
| Cronobacter | 38 | 12783 | 3653 |
| Cupriavidus | 22 | 33038 | 10572 |
| Curtobacterium | 6 | 7883 | 1851 |
| Cyanothece | 8 | 17167 | 5487 |
| Cycloclasticus | 4 | 2938 | 298 |
| Dehalobacter | 5 | 7912 | 2063 |
| Dehalococcoides | 12 | 2670 | 557 |
| Deinococcus | 23 | 24846 | 10163 |
| Delftia | 9 | 10294 | 1826 |
| Desulfitobacterium | 9 | 10985 | 2188 |
| Desulfobulbus | 11 | 14536 | 5823 |
| Desulfosporosinus | 8 | 16678 | 5264 |
| Desulfotomaculum | 12 | 20217 | 6556 |
| Desulfovibrio | 46 | 52986 | 18665 |
| Desulfurococcus | 4 | 2123 | 429 |
| Devosia | 4 | 9234 | 2308 |
| Dialister | 4 | 3684 | 834 |
| Dickeya | 38 | 14305 | 4694 |
| Dokdonia | 4 | 3853 | 481 |
| Dorea | 6 | 10262 | 3152 |
| Dyadobacter | 5 | 11854 | 3643 |
| Dyella | 4 | 8763 | 2536 |
| Edwardsiella | 17 | 7127 | 1554 |
| Eggerthella | 5 | 4781 | 920 |
| Ehrlichia | 16 | 2515 | 762 |
| Elizabethkingia | 15 | 7451 | 1835 |
| Endozoicomonas | 4 | 10461 | 3320 |
| Ensifer | 9 | 19131 | 6080 |
| Enterobacter | 158 | 34356 | 10355 |
| Enterococcus | 690 | 52806 | 16076 |
| Enterovibrio | 7 | 8800 | 1983 |
| Entomoplasma | 4 | 3907 | 2335 |
| Erwinia | 23 | 16991 | 6492 |
| Erysipelatoclostridium | 5 | 9631 | 2889 |
| Erythrobacter | 7 | 8385 | 2175 |
| Escherichia | 2707 | 57095 | 18254 |
| Eubacterium | 21 | 31621 | 11005 |
| Exiguobacterium | 24 | 11444 | 2230 |
| Facklamia | 5 | 4102 | 1093 |
| Faecalibacterium | 5 | 5686 | 1452 |
| Ferrimonas | 4 | 7927 | 1785 |
| Finegoldia | 6 | 2729 | 299 |
| Fischerella | 7 | 16643 | 6930 |
| Flavobacterium | 64 | 41164 | 17909 |
| Francisella | 65 | 5643 | 1307 |
| Frankia | 16 | 30081 | 9138 |
| Fusobacterium | 75 | 17352 | 4714 |
| Gallibacterium | 23 | 4631 | 749 |
| Gardnerella | 36 | 3828 | 964 |
| Gemella | 7 | 3470 | 767 |
| Geobacillus | 31 | 14741 | 5394 |
| Geobacter | 14 | 17928 | 3504 |
| Gilliamella | 4 | 3863 | 1015 |
| Gillisia | 5 | 7131 | 1527 |
| Glaciecola | 11 | 17288 | 5390 |
| Gluconobacter | 10 | 7176 | 2065 |
| Glycomyces | 4 | 8476 | 2310 |
| Gordonia | 31 | 32784 | 11082 |
| Granulibacter | 4 | 2950 | 348 |
| Haemophilus | 76 | 11077 | 2612 |
| Hafnia | 4 | 6516 | 1238 |
| Haloarcula | 11 | 9509 | 2836 |
| Halobacillus | 7 | 9631 | 3779 |
| Halococcus | 7 | 9910 | 3388 |
| Haloferax | 20 | 11915 | 3118 |
| Halomonas | 34 | 26607 | 9209 |
| Halopiger | 4 | 8032 | 2928 |
| Haloquadratum | 5 | 8071 | 3993 |
| Halorubrum | 21 | 13640 | 4815 |
| Haloterrigena | 6 | 9651 | 2962 |
| Helicobacter | 462 | 29453 | 13274 |
| Herbaspirillum | 15 | 19194 | 6202 |
| Hippea | 4 | 2984 | 715 |
| Hydrogenobaculum | 5 | 1725 | 72 |
| Hymenobacter | 6 | 10777 | 3539 |
| Hyphomicrobium | 6 | 12126 | 3936 |
| Hyphomonas | 13 | 13447 | 3522 |
| Idiomarina | 9 | 7532 | 2233 |
| Janthinobacterium | 7 | 15085 | 3783 |
| Jeotgalibacillus | 4 | 8539 | 3283 |
| Kinetoplastibacterium | 7 | 1210 | 234 |
| Kingella | 47 | 7351 | 1744 |
| Kitasatospora | 11 | 25875 | 8206 |
| Klebsiella | 414 | 29537 | 8830 |
| Knoellia | 4 | 6247 | 1314 |
| Kocuria | 9 | 9195 | 2657 |
| Komagataeibacter | 11 | 11005 | 4407 |
| Kosakonia | 4 | 6931 | 1249 |
| Kurthia | 4 | 5359 | 1652 |
| Labrenzia | 5 | 13011 | 3463 |
| Lachnoanaerobaculum | 4 | 4625 | 886 |
| Lachnoclostridium | 41 | 49094 | 16900 |
| Lactobacillus | 307 | 68388 | 22558 |
| Lactococcus | 51 | 12333 | 3766 |
| Lebetimonas | 6 | 2263 | 373 |
| Legionella | 45 | 22765 | 10792 |
| Leifsonia | 6 | 9123 | 2211 |
| Leisingera | 13 | 12140 | 3429 |
| Leptolyngbya | 8 | 24291 | 10962 |
| Leptospira | 322 | 53260 | 22712 |
| Leptospirillum | 7 | 10261 | 3571 |
| Leptotrichia | 12 | 6905 | 1828 |
| Leucobacter | 6 | 7730 | 2141 |
| Leuconostoc | 29 | 8323 | 2729 |
| Listeria | 124 | 17436 | 6624 |
| Loktanella | 4 | 7247 | 1293 |
| Luteibacter | 4 | 5379 | 1032 |
| Lysinibacillus | 18 | 17743 | 6304 |
| Lysobacter | 9 | 12871 | 4531 |
| Magnetospirillum | 4 | 11431 | 2890 |
| Mannheimia | 23 | 4526 | 735 |
| Maribacter | 4 | 8052 | 1921 |
| Marinobacter | 21 | 18366 | 6279 |
| Marinobacterium | 5 | 11587 | 3494 |
| Marinomonas | 6 | 10720 | 3132 |
| Massilia | 8 | 14089 | 3850 |
| Megamonas | 4 | 3678 | 1068 |
| Megasphaera | 11 | 6113 | 1749 |
| Meiothermus | 9 | 8182 | 1759 |
| Mesoplasma | 7 | 2805 | 1367 |
| Mesorhizobium | 80 | 52990 | 20636 |
| Metallosphaera | 4 | 3897 | 1178 |
| Methanobacterium | 9 | 7920 | 3056 |
| Methanobrevibacter | 30 | 6209 | 2181 |
| Methanocaldococcus | 8 | 3393 | 935 |
| Methanococcus | 10 | 4451 | 749 |
| Methanosarcina | 5 | 8272 | 2504 |
| Methylibium | 6 | 13831 | 3305 |
| Methylobacter | 9 | 12875 | 4350 |
| Methylobacterium | 24 | 32809 | 10176 |
| Methylocystis | 6 | 11223 | 4050 |
| Methylomicrobium | 4 | 7816 | 1450 |
| Methylomonas | 5 | 9875 | 2620 |
| Methylophaga | 6 | 6851 | 1710 |
| Methylophilus | 6 | 4293 | 778 |
| Methylosinus | 4 | 8608 | 2650 |
| Methylotenera | 10 | 7201 | 1832 |
| Methyloversatilis | 6 | 5967 | 885 |
| Microbacterium | 38 | 31183 | 9500 |
| Microbispora | 5 | 16089 | 4508 |
| Micrococcus | 5 | 8210 | 1266 |
| Microcystis | 16 | 11639 | 3078 |
| Micromonospora | 10 | 16143 | 2980 |
| Mobiluncus | 8 | 4400 | 919 |
| Moraxella | 4 | 5435 | 2277 |
| Morganella | 12 | 10351 | 2385 |
| Mycobacterium | 2520 | 119959 | 42602 |
| Mycoplasma | 150 | 26560 | 10105 |
| Myroides | 12 | 8529 | 2414 |
| Myxococcus | 5 | 11666 | 2448 |
| Natrialba | 7 | 9875 | 2670 |
| Natrinema | 9 | 8608 | 2638 |
| Natronorubrum | 4 | 6830 | 2228 |
| Neisseria | 246 | 18951 | 8048 |
| Nesterenkonia | 4 | 5423 | 1471 |
| Nitratireductor | 5 | 9909 | 2741 |
| Nitrosococcus | 6 | 6527 | 2204 |
| Nitrosomonas | 5 | 8113 | 2759 |
| Nitrosopumilus | 6 | 3708 | 1274 |
| Nitrosospira | 4 | 6493 | 2483 |
| Nitrospina | 4 | 5903 | 2513 |
| Nocardia | 42 | 67676 | 26173 |
| Nocardioides | 12 | 19112 | 5375 |
| Nocardiopsis | 21 | 27824 | 8817 |
| Nonlabens | 8 | 7371 | 1611 |
| Novosphingobium | 14 | 24110 | 8280 |
| Oceanicola | 7 | 12992 | 3659 |
| Oceanobacillus | 7 | 12502 | 3946 |
| Ochrobactrum | 11 | 11840 | 3034 |
| Oenococcus | 53 | 4832 | 1276 |
| Olsenella | 4 | 4335 | 1264 |
| Oribacterium | 10 | 10734 | 3029 |
| Oscillibacter | 6 | 9157 | 3290 |
| Paenibacillus | 109 | 134484 | 51862 |
| Pandoraea | 9 | 11329 | 3009 |
| Pantoea | 42 | 23000 | 8005 |
| Parabacteroides | 21 | 16541 | 4589 |
| Paracoccus | 19 | 18757 | 5162 |
| Parvimonas | 5 | 2275 | 318 |
| Pasteurella | 27 | 5787 | 1521 |
| Pectobacterium | 24 | 12741 | 3467 |
| Pediococcus | 11 | 4310 | 1064 |
| Pedobacter | 13 | 23764 | 9714 |
| Pelosinus | 8 | 8257 | 2396 |
| Peptoclostridium | 257 | 30036 | 9403 |
| Peptoniphilus | 17 | 10930 | 2845 |
| Peptostreptococcus | 4 | 3292 | 684 |
| Phaeobacter | 6 | 6453 | 1165 |
| Phascolarctobacterium | 4 | 4358 | 1344 |
| Photobacterium | 15 | 23057 | 7511 |
| Photorhabdus | 11 | 11102 | 3687 |
| Piscirickettsia | 6 | 3815 | 887 |
| Planktothrix | 8 | 6880 | 1403 |
| Planococcus | 5 | 6944 | 2121 |
| Polaribacter | 6 | 7592 | 1851 |
| Polaromonas | 5 | 12072 | 2859 |
| Pontibacillus | 5 | 9464 | 3594 |
| Porphyromonas | 58 | 23626 | 7788 |
| Prevotella | 108 | 54801 | 22765 |
| Prochlorococcus | 147 | 19916 | 7899 |
| Propionibacterium | 130 | 18799 | 5183 |
| Proteus | 10 | 6553 | 1560 |
| Providencia | 19 | 12303 | 3519 |
| Pseudoalteromonas | 57 | 38754 | 13533 |
| Pseudobutyrivibrio | 5 | 5539 | 1421 |
| Pseudogulbenkiania | 4 | 7549 | 1073 |
| Pseudomonas | 1143 | 147476 | 50889 |
| Pseudonocardia | 6 | 22637 | 6154 |
| Pseudoxanthomonas | 8 | 6725 | 1093 |
| Psychrobacter | 19 | 9981 | 3563 |
| Psychromonas | 7 | 12523 | 3952 |
| Psychroserpens | 5 | 7507 | 2128 |
| Pyrobaculum | 7 | 6327 | 1538 |
| Pyrococcus | 7 | 4294 | 727 |
| Rahnella | 6 | 6739 | 728 |
| Ralstonia | 35 | 24171 | 5827 |
| Raoultella | 4 | 6565 | 842 |
| Rheinheimera | 5 | 9534 | 3216 |
| Rhizobium | 108 | 85571 | 35997 |
| Rhodanobacter | 8 | 8513 | 2222 |
| Rhodobacter | 15 | 13280 | 2554 |
| Rhodococcus | 69 | 68248 | 21295 |
| Rhodopirellula | 9 | 24772 | 12052 |
| Rhodopseudomonas | 9 | 13323 | 3640 |
| Rhodospirillum | 4 | 7927 | 1838 |
| Rickettsia | 66 | 6435 | 2143 |
| Riemerella | 8 | 3352 | 657 |
| Roseburia | 5 | 7606 | 1721 |
| Roseobacter | 7 | 13406 | 3611 |
| Roseomonas | 5 | 10017 | 2655 |
| Roseovarius | 5 | 8347 | 1751 |
| Rothia | 9 | 4405 | 749 |
| Rubrivivax | 4 | 5489 | 467 |
| Ruegeria | 10 | 15282 | 4625 |
| Ruminiclostridium | 24 | 29766 | 10549 |
| Ruminococcus | 26 | 31810 | 11961 |
| Saccharomonospora | 12 | 14151 | 3694 |
| Saccharopolyspora | 7 | 15590 | 5004 |
| Salinicoccus | 4 | 4508 | 916 |
| Salinispora | 97 | 23297 | 6155 |
| Salinivibrio | 4 | 5473 | 1588 |
| Salmonella | 984 | 28177 | 9882 |
| Sediminibacterium | 4 | 6235 | 1347 |
| Selenomonas | 24 | 14635 | 4223 |
| Serratia | 61 | 26776 | 8722 |
| Shewanella | 45 | 30761 | 11198 |
| Shigella | 136 | 12293 | 2911 |
| Sinorhizobium | 45 | 28258 | 9732 |
| Slackia | 4 | 4713 | 893 |
| Smithella | 5 | 6976 | 3041 |
| Snodgrassella | 7 | 4196 | 1332 |
| Sodalis | 4 | 7442 | 1977 |
| Sphingobacterium | 11 | 23777 | 9320 |
| Sphingobium | 26 | 26061 | 9228 |
| Sphingomonas | 48 | 46126 | 16024 |
| Sphingopyxis | 7 | 9612 | 2703 |
| Spirochaeta | 9 | 17588 | 5842 |
| Spiroplasma | 12 | 5109 | 2139 |
| Spirosoma | 4 | 12699 | 3564 |
| Sporolactobacillus | 5 | 7344 | 2709 |
| Sporosarcina | 7 | 11731 | 3397 |
| Staphylococcus | 4320 | 28903 | 7519 |
| Stenotrophomonas | 26 | 12553 | 3184 |
| Streptacidiphilus | 10 | 26057 | 7687 |
| Streptococcus | 4514 | 82768 | 26477 |
| Streptomyces | 451 | 247449 | 63400 |
| Sulfitobacter | 14 | 14441 | 3664 |
| Sulfobacillus | 5 | 6107 | 1199 |
| Sulfolobus | 23 | 8171 | 1767 |
| Sulfuricurvum | 4 | 4843 | 1178 |
| Sulfurihydrogenibium | 4 | 2843 | 158 |
| Sulfurimonas | 4 | 4717 | 1164 |
| Sulfurospirillum | 8 | 6870 | 1746 |
| Sulfurovum | 7 | 8402 | 2990 |
| Sutterella | 4 | 4590 | 1229 |
| Synechococcus | 32 | 32753 | 14811 |
| Synechocystis | 6 | 7932 | 2331 |
| Tannerella | 7 | 7208 | 1963 |
| Tatumella | 4 | 6517 | 1933 |
| Taylorella | 21 | 2144 | 242 |
| Teredinibacter | 9 | 7606 | 1833 |
| Thalassospira | 6 | 8362 | 2064 |
| Thauera | 10 | 14891 | 3013 |
| Thermoanaerobacter | 17 | 6264 | 1385 |
| Thermoanaerobacterium | 6 | 4395 | 710 |
| Thermococcus | 14 | 7925 | 2165 |
| Thermodesulfobacterium | 6 | 3231 | 617 |
| Thermotoga | 23 | 6834 | 1154 |
| Thermus | 22 | 6913 | 1522 |
| Thioalkalivibrio | 74 | 21477 | 5682 |
| Thioclava | 4 | 7234 | 1673 |
| Thiomicrospira | 10 | 8110 | 2791 |
| Thiothrix | 4 | 8392 | 2020 |
| Treponema | 56 | 31913 | 10941 |
| Tropheryma | 18 | 1246 | 209 |
| Ureaplasma | 20 | 1638 | 452 |
| Variovorax | 19 | 18017 | 4934 |
| Veillonella | 22 | 6738 | 1976 |
| Verrucomicrobium | 4 | 11306 | 2734 |
| Vibrio | 676 | 98225 | 36394 |
| Virgibacillus | 7 | 13760 | 3984 |
| Weissella | 12 | 6225 | 1798 |
| Wolbachia | 26 | 4268 | 1888 |
| Xanthomonas | 207 | 29153 | 10222 |
| Xenorhabdus | 17 | 12551 | 4434 |
| Xylella | 16 | 6557 | 2537 |
| Yersinia | 174 | 20378 | 7128 |
| Zymomonas | 7 | 3024 | 639 |
