# Supplementary material for: PATtyFams: Protein Families for the Microbial Genomes in the PATRIC Database
Source: Front Microbiol. 2016 Feb 8;7:118. doi: 10.3389/fmicb.2016.00118 (PMC4744870; doi:10.3389/fmicb.2016.00118)
Supplement: Supplementary file 5 [file Table5.DOCX]

**Table S5.** The set of core protein families created by each protein family generation method. Core families are defined as those containing a protein from ≥ 90% of the genomes in each set.

| Genome sets | FIGfams | kClust | OrthoMCL | PATtyFams |
| --- | --- | --- | --- | --- |
| Brucella (43 genomes) |  |  |  |  |
| Total Families | 2,232 | 2,459 | 2,991 | 2,984 |
| Total Proteins | 131,155 | 110,320 | 131,039 | 133,522 |
|  |  |  |  |  |
| Escherichia (38 genomes) |  |  |  |  |
| Total Families | 2,598 | 2,705 | 3,100 | 3,044 |
| Total Proteins | 122,496 | 112,204 | 119,434 | 116,726 |
|  |  |  |  |  |
| Global Families (80 diverse genomes) |  |  |  |  |
| Total Families | 219 | 23 | 248 | 188 |
| Total Proteins | 45,527 | 1,840 | 20,820 | 15,759 |
